# Comparative Analysis of Thermal Behavior, Isothermal Crystallization Kinetics and Polymorphism of Palm Oil Fractions

**DOI:** 10.3390/molecules18011036

**Published:** 2013-01-15

**Authors:** Xia Zhang, Lin Li, He Xie, Zhili Liang, Jianyu Su, Guoqin Liu, Bing Li

**Affiliations:** 1 College of Light Industry and Food Sciences, South China University of Technology, Guangzhou 510640, Guangdong, China; E-Mails: z.xia.scut@gmail.com (X.Z.); felinli@scut.edu.cn (L.L.); heige_2011@yahoo.cn (H.X.); liangzhili02@163.com (Z.L.); jysu@scut.edu.cn (J.S.); hnut2005@163.com (G.L.); 2 Guangdong Province Key Laboratory for Green Processing Of Natural Products and Product Safety, Guangzhou 510640, Guangdong, China

**Keywords:** fractional crystallization, thermal behavior, isothermal crystallization kinetics, polymorphism

## Abstract

Thermal behavior of palm stearin (PS) and palm olein (PO) was explored by monitoring peak temperature transitions by differential scanning calorimetry (DSC). The fatty acid composition (FAC), isothermal crystallization kinetics studied by pulsed Nuclear Magnetic Resonance (pNMR) and isothermal microstructure were also compared. The results indicated that the fatty acid composition had an important influence on the crystallization process. PS and PO both exhibited more multiple endotherms than exotherms which showed irregular peak shapes. An increasing in cooling rate, generally, was associated with an increase in peak size. Application of the Avaimi equation to isothermal crystallization of PS and PO revealed different nucleation and growth mechanisms based on the Avrami exponents. PS quickly reached the end of crystallization because of more saturated triacylglycerol (TAG). The Avrami index of PS were the same as PO under the same isothermal condition at lower temperatrue, indicating that the crystallization mechanism of the two samples based on super-cooling state were the same. According to the polarized light microscope (PLM) images, crystal morphology of PS and PO was different. With the temperature increased, the structure of crystal network of both PS and PO gradually loosened.

## 1. Introduction

Palm oil is extracted from the mesocarp of the fruit of the oil palm *Elaeis guineensis*. It is semi-solid and high in saturated fat at room temperature, with a natural melting point of about 306 K. Palm oil exhibits several excellent properties such as high productivity, low price, ease of the formation of β' type crystal, high thermal oxidative stability and plasticity at room temperature conditions [1]. Therefore, palm oil is considered to be the most economical and abundant edible oil worldwide in the near future [2] and has been widely used as cooking oil, margarine, shortening in cooking, confectionery, bakery *etc.* However, the application of palm oils in their original form is limited due to their specific chemical composition. Palm oil consists of high melting point triacylglycerols (TAGs) and low melting point TAGs, resulting in a broad melting range. In practice, the modification operation of hydrogenation or interesterification (chemical or enzymatic) or fractionation on the physicochemical properties of palm oil would extend its use in food industry. Among these modification methods, dry fractionation is a fully reversible modification and is the simplest and cheapest fractional crystallization processes, due to the absence of effluent, no chemical consumption and no losses [3]. In the dry fractionation process, according to the differences in melting points of TAGs, palm oil can be fractionated into many components containing liquid fractions (oleins, super oleins and top oleins) which can be used as cooking and salad oils, and harder fractions (stearins and mid fractions) which have applications as ingredients of frying fats, margarines, shortenings, as well as specialty fats [4]. With the increasing health questions about *trans* fatty acids, replacement of partially hydrogenated fats by palm oils and palm oil fractions has attracted more and more attention. Palm stearin and palm olein manufactured by dry fractionation have different triacylglycerol compositions, thus resulting in oil-based margarine and shortening to present complex crystallization behaviors and different physical properties. Busfield and Proschogo observed several thermal transitions of palm stearin within their differential scanning calorimetry (DSC) profiles [5]. Braipson-Danthine and Gibonb pointed out that there was a clear relationship between TAG composition, melting properties and polymorphic behavior and of palm oil and fractions after analyzing a series of palm oil, solid and liquid fractions (stearins, mid fractions, oleins and superoleins) [6]. Zaliha *et al.* studied the crystallization of palm oil in relation to the dry fractionation process, and found that the physical properties of the fractions were dependent on the cooling sequence [7]. Using polarized light microscope (PLM) and X-ray diffraction (XRD), Chen *et al.*,found that during two-stage dry fractionation process of palm oil at temperatures below 295 K, the spherical crystals formed from the first fraction were in α form, whereas the needle-like crystals that nucleated later from the second fraction were in β’ form [8]. Palm oil fractions originated from different dry fractionation operation conditions exhibit different TAG composition and physical properties, moreover, crystallization behaviors of palm oil fractionations will also affect the physicochemical properties of the end products such as margarine and shortening, therefore it is of great significance to explore the crystallization behavior of the palm oil fractions. 

Palm stearin (PS) of iodine value (IV) 8 and palm olein (PO) of iodine value (IV) 48 are the elementary palm oil fractions, which along with palm oil are the base oil when mixing to produce a lot of palm oil mixture products with different melting points and iodine values being applicable in many kinds of foods.

In this study, in order to gain a better understanding of the isothermal kinetics of PS and PO, the thermal behavior of PS and PO was investigated using DSC, and their isothermal crystallization kinetics was compared by pulsed Nuclear Magnetic Resonance (pNMR). From the isothermal crystallization kinetics, the crystallization process and the mechanisms of crystal forming and growth can be described in detail [8], which are very important for the quality of the end products. Isothermal crystallization exotherms obtained in this work were used to calculate the kinetic parameters according to the Avrami equation. The Avrami model was used primarily in the determination of *n*, the exponent in the Avrami equation, the value of which is associated with the mechanisms of crystal growth. A crystallization process in homogeneous nucleation with *n* = 4 follows a polyhedral crystal growth mechanism, a value of n = 3 a plate-like crystal growth mechanism, and n = 2, a linear crystal growth. Noninteger values of n are associated with heterogeneous and secondary nucleation [9]. Also, their non-isothermal crystallization respectively at a cooling rate of 1, 5, 10 and 20 °C/min was tested.

## 2. Results and Discussion

### 2.1. Fatty Acid Composition (FAC)

The fatty acid composition of PS and PO is shown in Table 1.

**Table 1:** Fatty acid composition of PS and PO (%).

Fatty acids	C_10:0_	C_12:0_	C_14:0_	C_16:0_	C_18:0_	C_18:1_	C_18:2_	other
Palm stearin	5.00 ± 0.13	2.79 ± 0.16	6.63 ± 0.25	42.24 ± 0.34	4.46 ± 0.09	30.39 ± 0.52	5.99 ± 0.31	2.50 ± 0.08
Palm olein	5.27 ± 0.18	2.73 ± 0.11	5.33 ± 0.19	31.18 ± 0.26	3.89 ± 0.14	39.42 ± 0.27	9.76 ± 0.44	2.42 ± 0.11

As indicated in Table 1, PS and PO had the same kinds of fatty acids, but were different in content. PS was high in saturated fatty acid content, while PO was high in unsaturated fatty acid content. The content of C_16:0_ and C_18:0_ accounted for 46.70% fatty acids in PS, the content of C_16:0_ in PS was 26.18% higher than that in PO. PO contained 49.18% unsaturated fatty acid (C18:1 and C_18:2_), the contents of C_18:1_ and C_18:2_ in PO were respectively 22.90% and 38.62% higher than those in PS. The composition difference was attributed to the dry fractionation process where as crystallization proceeds, the more saturated triglycerides are gradually concentrated in the solid phase (stearin), leaving behind a more unsaturated liquid phase (olein) [7]. Contents of the fatty acids composition matched with the iodine values of PS and PO. Higher iodine value was related with more unsaturated acid. Moreover, both PS and PO contained a small amount of carbon-chain fatty acids which made TAG compositions more complex. Generally, as a mixture of TAGs, natures of oil were closely related to the compositions [10]. As a result, the thermal behavior of PS and PO will be closely related to their compositions.

### 2.2. Crystallization and Melting Behavior

**Figure 1:**
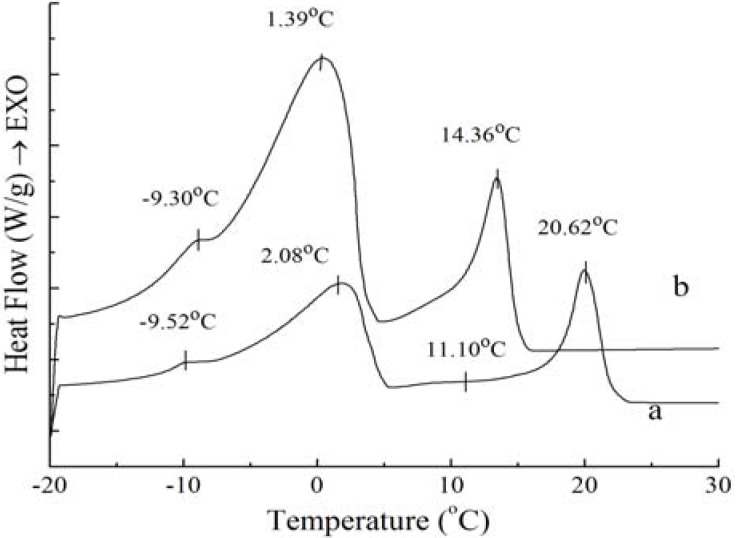
Crystallization curves of PS (**a**) and PO (**b**).

**Figure 2:**
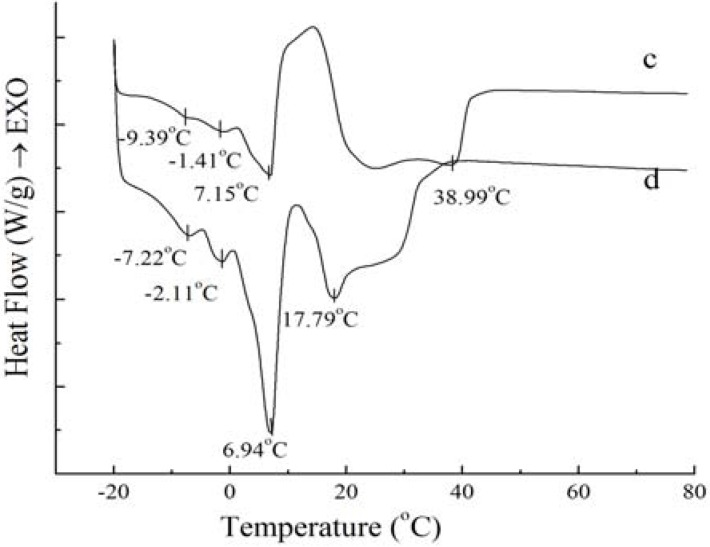
Melting curves of PS (**c**) and PO (**d**).

The most important aspect of the physical properties of oils and fats is related to their melting and crystallization behavior [11]. If crystallization conditions changed, crystal habit, crystal size and crystal numbers would be influenced. These changes will eventually be reflected in the product performance. In order to study the physical properties of PS and PO, their crystallization and melting behaviors were analyzed by DSC and shown in Figure 1 and Figure 2.

As Figure 1 shows, exothermic peak changes appear during the crystallization process, which may be due to polymorphic changes. In the crystallization profiles for PS and PO samples, three similar exotherms emerged, indicating that these two samples had similar crystallization thermodynamics. The crystallization profile is characterized by the beginning of fat crystal formation, which is related with the rearrangement of molecules due to the presence of high saturated TAGs, and the end of crystallization, which is generally reflected by aggregation and compaction of molecules [11]. The peak temperature and the required enthalpy variations of crystallization thermograms of PS and PO with 5 °C/min have been calculated, the exotherms temperature and enthalpy variations of the four peaks of PS are −9.52 ± 0.08 °C and 0.37 ± 0.10 J/g, 2.08 ± 0.08 °C and 21.93 ± 0.37 J/g, 11.10 ± 0.06 °C and 0.12 ± 0.01 J/g, 20.62 ± 0.44 °C and 15.76 ± 0.42 J/g, when those of PO come to −9.30 ± 0.12 °C and 0.34 ± 0.09 J/g, 1.39 ± 0.11 °C and 19.12 ± 0.32 J/g, 14.36 ± 0.35 °C and 8.51 ± 0.32 J/g. Exothermic peaks of PO were sharper compared to those of PS; this was due to the fact that the TAG distribution of PO was not as broad as that of PS. The temperatures and enthalpy variations of the two main exothermic peaks of PS were higher than those of PO. In essence, as the melt is cooled from above the crystallization temperature to below the crystallization temperature, it can be explained by the reason that there are more higher melting components in PS and more less melting components in PO. It can clearly be seen that the higher melting component exhibit more rapid crystallization. These results are in accordance with those reported for palm oil samples [12]. Tan and Che Man [11] and Saadi *et al.* [13] have reported that the exothermic thermogram was affected only by the chemical composition of the oil and not by primary crystallization state, and the changes in the temperature transition of cooling thermograms display the behaviour of triacylglycerols contents as principal feature. The peak temperature of SUS(S- saturate fatty acid; U- unsaturated fatty acid)TAGs and SSS TAGs was −11.22~−1.69 °C and 13.72~27.64 °C when the cooling rate was 5 °C /min for the crystallization process studied by Che Man [12]. According to Figure 1, it can be obtained that the exothermic peaks of PS and PO corresponded with those for SSS TAGs and SUS TAGs. 

Figure 2 shows that the melting curves are more complicated than the crystallization curves, being broad and overlapping. The broadening effects between the four peaks were due to the broad TAG distribution of the samples [14]. The melting peaks are multiple and irregular with four endotherms in Figure 2. According to Fredrick *et al.* [15], the melting profiles gave an indication of the amount of crystallized fat and the occurrence of polymorphic transitions. For Figure 2, the complexity of the melting curves shows there are different amount of crystallized fat and several types of homogeneous polycrystalline of PS and PO. The peak temperature and the required enthalpy variations of melting thermograms of PS and PO with 5 °C/min have been calculated. The peak temperature and the enthalpy variations of PS are −9.39 ± 0.12 °C and 0.19 ± 0.05 J/g, −1.41 ± 0.14 °C and 0.69 ± 0.08 J/g, 7.15 ± 0.12 °C and 13.06 ± 0.21 J/g, 38.99 ± 0.35 °C and 67.61 ± 0.48 J/g , while those of PO come to −7.22 ± 0.18 °C and 0.50 ± 0.11 J/g, −2.11 ± 0.09 °C and 0.66 ± 0.10 J/g, 6.94 ± 0.07 °C and 10.53 ± 0.24 J/g, 17.79 ± 0.11 °C and 14.66 ± 0.18 J/g. According to Kawamura [9], the different type of homogeneous polycrystalline presented with different enthalpy variations. Garti *et al.* [16] studied the role of emulsifier on the stabilization of the β' form and found that the first peak of the heating thermogram corresponded to the melting of the α form, while the last peak corresponded to the melting of the β form, and the intermediate peak indicated the melting of the β' form. Also, Che Man and Swe [17] found that the low-temperature peaks represented polymorphs β_2_' and α, while the high-temperature peaks represented polymorphs β_1_' and β_1_ according to the heating curves of refined, bleached and deodorized palm oil. Busfield and Proschogo [5] analyzed from the heating thermogram that the structures of palm stearin were α, β', and β. Che Man [12] have studied the heating thermogram of palm oil and palm stearin and pointed out that the peaks of PS around 39.2 °C and 6.9 °C represented polymorphs β_1_’ and α, the peaks of PO around 19.4 °C and 6.2 °C represented polymorphs β_1_' and α, and the other peaks at the temperature below 0 °C could not be confirmed the polymorphs. Consequently, it can be proved that there were different types of homogeneous polycrystalline PS and PO for this study.

Figure 2 also demonstrates that the endothermic peaks of PO were sharper compared to those of PS. When the temperature was lower than 10 °C, the endothermic peaks of PO were larger than those of PS, indicating that there was more low-melting fatty acids in PO. When the temperature ranged from 17 °C to 40 °C, the endothermic peaks of PS were larger than those of PO, which was consistent with the higher content of high melting crystallized fat in PS.

### 2.3. Thermal Behavior with Different Cooling Rates

**Figure 3:**
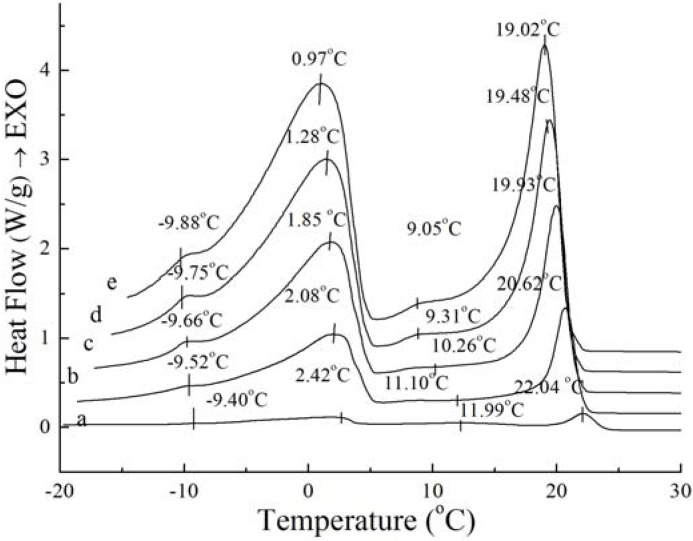
Crystallization curves with different cooling rate of PS: 1 °C/min (**a**), 5 °C/min (**b**), 10 °C/min (**c**), 15 °C/min (**d**), 20 °C/min (**e**).

**Figure 4:**
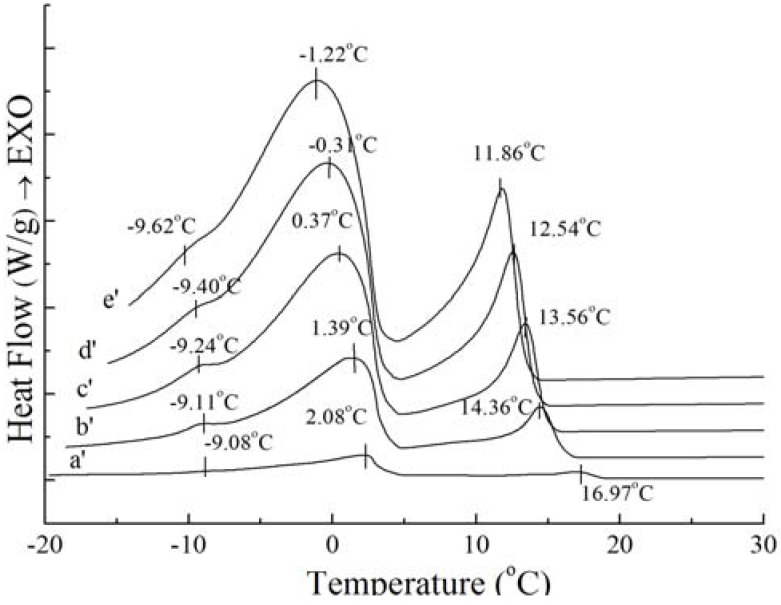
Crystallization curves with different cooling rate of PO: 1 °C/min (**a'**), 5 °C/min (**b'**), 10 °C/min (**c'**), 15 °C/min (**d'**), 20 °C/min (**e'**).

DSC crystallization profiles and data under different cooling rates are summarized in Figure 3 and Figure 4. The scanning rate was 1, 5, 10, 15, 20 °C/min, respectively. The curves in Figure 3 are similar with those in Figure 4, so it can be considered that PS and PO have similar crystallization profiles changing with the cooling rate. From the figures, it can be seen that the position of the exotherms is dependent on the cooling rate, while the amount of the exotherms is independent on the cooling rate. The crystallization exotherms came to lower temperature as the cooling rates increasing. Also, the crystallization exotherms got broadened with the cooling rate increasing. For PS, two small exothermic peaks and two big sharp exothermic peaks can be seen in the range of 1 °C/min to 20 °C/min, when there is one small exothermic peak and two big sharp exothermic peaks in PO (Figure 3 and Figure 4), it may be caused by the dependence of the heat history of crystallization process. These results are in accordance with the study reported for milk fat and lard [18]. It may correspond to the differential crystallization of higher melting TAGs firstly and lower melting TAGs secondly. When the crystallization process is slow, TAGs of similar chain lengths have time to associate with each other, co-crystallize, and fractionate. As the cooling rate increases, so does the rate of crystallization. As the system is crystallized rapidly, higher melting TAGs will be rapidly undercooled and initially crystallized, developing a solid within the liquid phase [19], and the polymer chains which come into crystalline lattice require a certain “relaxation” time, resulting in a “lag period” compared to the cooling process and it increases with cooling rate increasing [20]. In addition, this is accompanied by a rapid increase in viscosity, thus heat transfer and mass transfer may be limited. Lower mobility of molecular chain prevented rearrangement of the crystal, thus forming unstable crystal. 

Although the amount and shape of exothermic peaks for PS and PO in crystallization curves at different cooling rates are similar, the height and width of these peaks are quite different between PS and PO. The peak temperature and the required enthalpy variations of the main two big sharp peaks of PS and PO with different cooling rate have been calculated in Table 2. As Table 2 shows, all of temperatures of the main two exothermic peaks of PS and enthalpy values were higher than those of PO at the same cooling rate. This meant that PS was more easily crystallized with existing saturated TAGs. Crystallization at slow cooling rates gave more time to allow interactions between TAGs. The observed exothermic peak was attributed to the crystallization from transition of the saturated TAG. For PS and PO, with the cooling rate decreased, crystallization temperature of the peaks shifted to higher temperature while the enthalpy values became lower. Both enthalpy values of PS and PO provide a good expression on enthalpy requirements during interactions. This event was possibly attributed to the saturated TAGs. 

**Table 2:** Peak temperature(Tp)and enthalpy variation(∆Hc) of PS and PO with different cooling rate.

Sample	Dc (°C/min)	Peak I		Peak II	
		Tp (°C)	△Hc (J/g)	Tp (°C)	∆Hc (J/g)
PS	1	22.04 ± 0.25	9.74 ± 0.65	2.42 ± 0.11	13.78 ± 0.23
	5	20.62 ± 0.44	15.76 ± 0.42	2.08 ± 0.08	21.93 ± 0.37
	10	19.93 ± 0.27	16.20 ± 0.12	1.85 ± 0.16	23.59 ± 0.35
	15	19.48 ± 0.11	16.18 ± 0.33	1.28 ± 0.04	24.93 ± 0.28
	20	19.02 ± 0.21	17.02 ± 0.35	0.97 ± 0.07	25.30 ± 0.19
PO	1	16.97 ± 0.28	4.78 ± 0.19	2.08 ± 0.05	11.47 ± 0.25
	5	14.36 ± 0.35	8.51 ± 0.32	1.39 ± 0.11	19.12 ± 0.32
	10	13.56 ± 0.24	8.53 ± 0.28	0.37 ± 0.04	19.73 ± 0.19
	15	12.54 ± 0.37	8.26 ± 0.15	−0.31 ± 0.03	21.68 ± 0.27
	20	11.86 ± 0.14	7.98 ± 0.22	−1.22 ± 0.10	24.26 ± 0.65

### 2.4. Isothermal Crystallization Curves by pNMR

**Figure 5:**
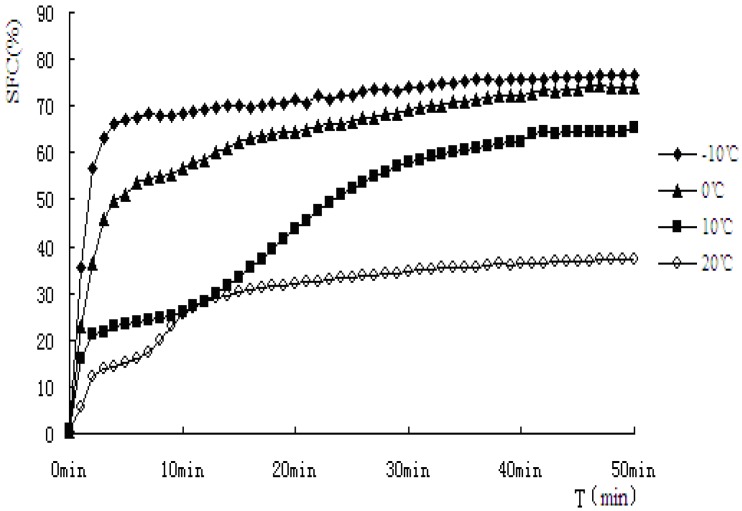
Solid fat content *vs.* time of crystallization for PS.

**Figure 6:**
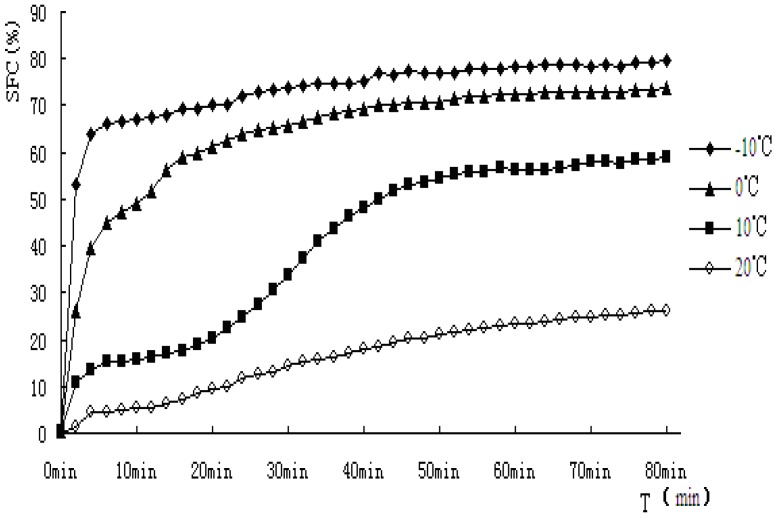
Solid fat content *vs.* time of crystallization for PO.

It can be seen the cooling rates have strong effects on the crystallization behavior of PS and PO from the above results, so different crystallization behavior at different temperatures was exhibited, and this may affect the quality of oil and its products. In this section, for further study of the crystallization mechanism of PS and PO at constant temperature, PS and PO were crystallized respectively at −10 °C, 0 °C, 10 °C and 20 °C isothermal crystallization curves of PS and PO were examined by pNMR and analyzed. Solid fat content *versus* time profile during isothermal crystallization of PS and PO under the temperature of −10 °C, 0 °C, 10 °C, 20 °C can be seen from Figure 5 and Figure 6. Noticeable differences in the shape of the curves of the component fats can be observed.

There are two crystallization steps, one is nucleation forming, and the other is crystal growth. In order to form crystal nucleii, the fat system should stay in super-cooling or as a supersaturated system. Once the nucleation forms, they continue to grow and form crystals [21]. Fat crystal generally experiences a fat-free period, nucleation form, rapid crystal accumulation period and endgame of crystallization with slowing down of speed and reaching the maximum. The shape of crystal SFC *vs.* time can give important clues for the crystallization mechanism of a particular substance [22]. In a study of the interaction of partial acylglycerols and triacylglycerols in milk fat, Foubert *et al.* [23] reported two mechanisms of interaction with the triacylgycerol crystals. The first resulted in slower crystal growth, due to competition in binding with the triacylglycerols. The second form involved organization in a micelle structure of partial acylglycerols, acting as nucleation facilitators. 

As indicated from the Figure 5 and Figure 6, when PS and PO stayed at lower temperature, higher SFC emerged. In this study, PS and PO were all heated up to 80 °C and held at this temperature for 20 min to clear the crystal history, then cooled to −10 °C, 0 °C, 10 °C, 20 °C for crystallization. Super-cooling level was higher when isothermal crystallization temperature was lower, so the nucleation rate exhibited rapid change as the crystallization rate did. Both the samples showed obvious S-curves at 10 °C and 20 °C, however, S-curves was not so obvious at −10 °C and 0 °C. The reasons were that nucleation and crystallization were at a faster rate when isothermal crystallization occurred at −10 °C and 0 °C. The fat-free period was inconspicuous, the fat could reach the end of crystallization quickly.

The crystallization curves of PS and PO show sharp slopes when the crystallization began at −10 °C and 0 °C. The slopes of PS and PO are almost the same. However, when the temperatures come to 10 °C and 20 °C, the slopes of PS were larger than those of PO, indicating crystallization rate of PS was higher. When the crystallization time came to 10 min, PS reached a stable crystalline form basically at −10 °C, 0 °C, 10 °C. Then the SFC changed very slowly, PO showed a slower changing rate at the same temperature. It may be due to the complex compositions of the samples. PS would crystallize faster and quickly come to the stable crystal form under the same crystallization temperature condition. At 20 °C, the SFC changing rates of PS and PO both slow down. 

Different isothermal crystallization curves signify different crystallization mechanisms. Temperature changing not only affected the solid fat content of products but also affected the crystallization behavior of the product. Thus it would affect the nature of the products. In order to describe the crystallization behavior in more detail, the Avrami crystallization theory has been used, the Avrami equation fitted with the percentage of crystallinity, as a function of time by linear regression. The equation fitted the data very well over the entire range of fractional crystallization values. Correlation coefficients obtained were always higher than 0.96 for the linear regression of log[−ln (1−X)] on log(t). The Avrami exponent (*n*), Avrami constant (*K*), and the half-times of crystallization (*t_1/2_*) determined from the curve fits has been calculated and the results were shown in Table 3.

**Table 3:** Isothermal crystallization kinetic parameters for PS and PO.

Samples	T	n	K/min^−n^	t_1/2_/min	R^2^
PS	−10 °C	0.4579 ± 0.0101	0.7976 ± 0.0021	0.7356 ± 0.1027	0.999
	0 °C	0.5708 ± 0.0098	0.4003 ± 0.0109	2.6156 ± 0.2319	0.998
	10 °C	0.8871 ± 0.0110	0.1032 ± 0.0099	8.5568 ± 0.3471	0.999
	20 °C	0.8591 ± 0.0087	0.1528 ± 0.0204	5.8117 ± 0.1827	0.996
PO	−10 °C	0.4089 ± 0.0203	0.7204 ± 0.0331	0.9095 ± 0.0218	0.998
	0 °C	0.6034 ± 0.0301	0.3420 ± 0.0278	3.2233 ± 0.1029	0.997
	10 °C	1.0943 ± 0.0189	0.0302 ± 0.0012	17.5173 ± 0.5621	0.999
	20 °C	1.1655 ± 0.0076	0.0174 ± 0.0026	23.6026 ± 0.4875	0.999

As it can be seen in Table 3, the Avrami exponent *n* for PS and PO increased with increasing crystallization temperature, however, at a set temperature, the difference between the values of *n* between PS and PO was slight. It meant that PS and PO have the same mechanism of nucleation and crystal growth. The value of *n* reflects the nucleation and growth mechanism for details, the high value of *n* means a more complex mechanism of crystal growth. Kawamura [9] has tabulated values for the Avrami exponent *n* for various types of nucleation and growth. The value ranges from 0.5 to 4. For example, an *n* of 4 indicates a polyhedral crystal growth mechanism, where an *n* of 3 represents a plate-like crystal growth mechanism, and an *n* of 2 indicates a linear crystal growth. The smaller the *n* value, the faster nucleation forms and grows. Wright *et al.* [24] also pointed out that the n value of 1 corresponded to rod-like growth from instantaneous nuclei, whereas spherulitic growth from sporadic nuclei is expected when a value of 4 is obtained. Litwinenko *et al.* [25] have found that margarine system was generally a heterogeneous nucleation when the Avrami exponent (n) generally increased with increasing temperature, from approximately 1 to 4. For Table 2, the value of *n* increases with the temperature rising indicated that the temperature changes the mechanism of nucleation, it turned to low super-cooling sporadic nucleation from the high super-cooling instantaneous nucleation with the temperature increasing. 

Besides, from the figures, apart from 20 °C of PS, the crystallization temperature increasing resulted in an decrease of crystallization rate constant(*K*) of PS and PO. This may correspond to the different driving forces of crystallization. The driving force depends on the super-cooling or super-saturation conditions, so the higher super-cooling was, the more rapidly crystallization happened. The crystallization would become slower with the lower super-cooling due to increasing temperature. The rate constant *K* of PS was higher than PO indicated that the crystallization rate of PS was higher than it of PO at the same temperature. The values of *K* and *n* had a significant change at 10 °C where *K* increased and *n* reduced significantly. This indicated that high temperature could greatly decrease the crystallization rate when the temperature was from −10 °C to 10 °C. 

The half-time of crystallization (*t_1/2_*) defined as the time which the extent of crystallization is 50% complete. The shorter the half-time was, the faster the crystallization rate obtained. As shown in Table 3, a higher temperature leads to a lower super-cooling degree, thus the nuclei for the crystallization had more difficulty to form. PS had more saturated TAG, so it would result in higher super-cooling and nucleation could occur easily, so the *t_1/2_* value of PS was lower than PO at the same temperature. 

### 2.5. Crystal Morphological Observation

The disagreement between the SFC and TAGs of PS and PO, together with their different nucleation and growth mechanisms at different temperatures, as discussed in the preceding sections, implies differences in the crystal morphologies of the two samples. The most popular method to visualize the microstructure of fat crystal network is PLM. For the food with high fat content, the strength of crystal network determines the 60–80% hardness [26]. After concluding the previous research of macroscopic physical properties of solid lipids, Narine *et al.* found that compared with other crystallization behaviors, the microstructure of fat is more significant to the macroscopic physical properties [27].

**Figure 7:**
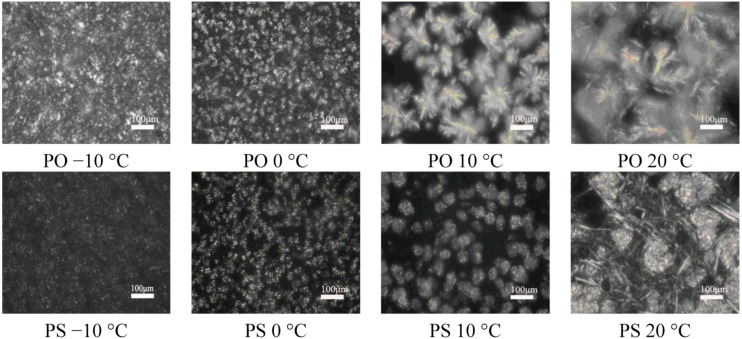
PLM images (×100) of PS and PO at −10 °C, 0 °C, 10 °C, 20 °C.

Figure 7 shows the isothermal photomicrographs of crystals of PS and PO at −10 °C, 0 °C, 10 °C and 20 °C. As indicated in the images, PS showed different crystal morphology compared to PO at the same temperature. Also, PS and PO formed different shapes of crystals at different temperatures. At −10 °C, both particles sizes of PS and PO are small, and well-distributed throughout the crystal system, and the crystals of PS were smaller than PO. Crystal aggregations can be seen in the photograph of PO while few were found in PS. At 0 °C, the number of spherical crystals of PS and PO were both decreased. PO formed a larger number of crystals and a denser network compared to PS, indicating that there were stronger interactions between the crystals of PO. When the temperature come to 10 °C, PO formed needle-like, clear frame crystals while PS showed spherical crystals and smaller particle sizes than that of PS. As to PS, the decreasing number of crystals indicated that the interaction between the crystals was reduced. When the temperature rises to 20 °C, disordered links between spherical crystals of PO were melted. Both of them showed independent, few, and larger size crystals, where PO still formed needle-like crystals and PS still formed spherical crystals. Summarily, a conclusion can be drawn that with the temperature increased, the structure of crystal network gradually loosened.

### 2.6. Melting Profile of PS and PO under Isothermal Crystallization

**Figure 8:**
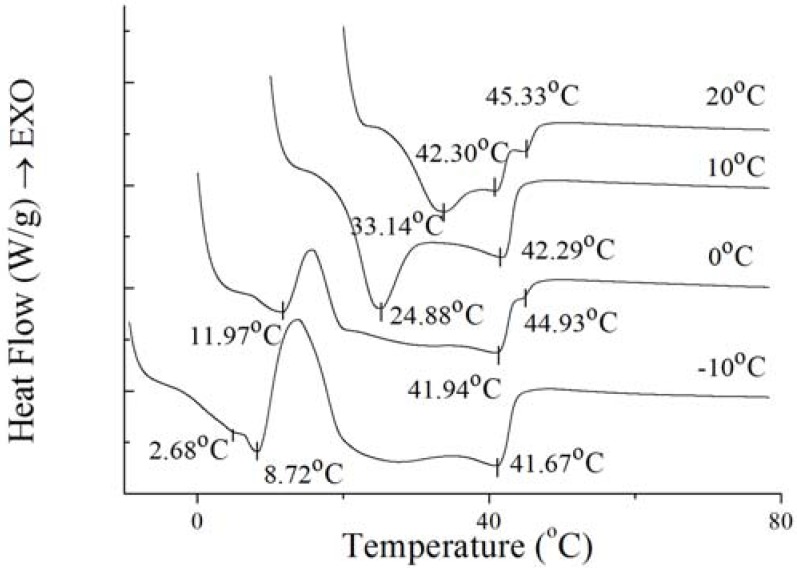
Melting thermogram at 5 °C/min on PS at the end of isothermal crystallization at various temperatures of crystallization.

**Figure 9:**
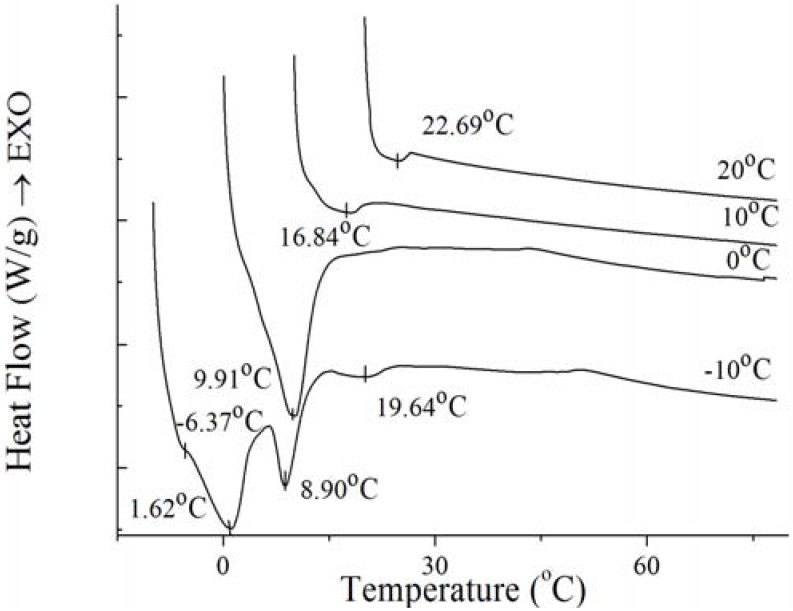
Melting thermogram at 5 °C/min on PO at the end of isothermal crystallization at various temperatures of crystallization.

To further investigate the nature of the crystals produced under isothermal crystallization, the melting profile of PS and PO was obtained by heating the sample at the end of each isothermal crystallization process. The results are shown in Figure 8 and Figure 9. According to Figure 8 and Figure 9, the crystals of PS formed at −10 °C showed a small low-melting endothermal peak around 2.68 °C, a sharp endothermal peak around 8.72 °C and a broad high-melting endothermal peak around 41.67 °C in the melting thermogram. At this temperature, fast nucleation with small spherulitic crystals was observed (Figure 7). The crystal structure was consistent with the growth mechanism which was presented above. For the size of the crystal of PS increased which formed at 0 °C, the low-melting endothermal peak with temperature of 2.68 °C disappeared and the endothermal peak around 8.72 °C and 41.94 °C decreased in size and increased in peak temperature. It can be interpreted by that there were more high-melting components in PS at higher temperature and different polymorph states were developed with different super-cooling. This is consistent with the crystal structure in Figure 7. Then with the temperature increasing to 10 °C, PS showed spherical crystals with increasing size (Figure 7), and the low-melting endothermal peaks disappeared and the higher melting peak showed an increase in its peak temperature (Figure 8). It indicated that a more stable polymorph state was developed [28]. When the temperature rises to 20 °C, the size of spherical crystals increased (Figure 7) with the peak temperarue of endothermal peaks increased (Figure 8). 

For the well-distributed small crystals of PO formed at −10 °C (Figure 7), there were two small endothermal peaks around −6.37 °C and 19.64 °C, two sharp endothermal peaks around 1.62 °C and 8.90 °C. When the temperature arrived 0 °C, there was only a big sharp endothermal peak around 9.91 °C while other peaks disappeared which appeared at 0 °C. At this temperature, small spherulitic crystals was observed in Figure 7. The needle-like, clear frame crystals formed at 10 °C (Figure 7) when there was only one small broaden endothermal peak around 16.84 °C in the melting thermogram. Then the size of this peak decreased and the temperature increased to 22.69 °C at 20 °C, melting spherical crystals of PO were presented (Figure 7). The complexity of the melting curve shows there are different amounts of crystallized fat and several types of homogeneous polycrystalline forms [9]. It can be concluded that with the temperature increased, the different types of homogeneous polycrystalline form were developed with different nucleation and growth mechanisms.

## 3. Experimental

### 3.1. Materials and Chemicals

PS with iodine value (IV) of 8 and melting point of 44 °C, and PO with IV of 48 and melting point of 24 °C were purchased from Yi Kerry Co. (Guangzhou, China). All chemicals used were of analytical or high-performance liquid chromatography (HPLC) grade. 

### 3.2. Fatty Acid Composition (FAC)

Fatty acid composition of palm oil fractions was determined through direct methylation with sodium methoxide and methanol. Fatty acid methyl esters were prepared according to the AOCS Official Method Ce 2-66 [29] and subsequently analyzed on a GC-14B gas chromatograph (GC) equipped with a fused silica capillary column (CP-Si188, 100 m × 0.25 mm × 0.2 mm i.d.) with a flow rate of 1 mL N_2_/min, and a flame ionization detector (Shimadzu, Tokyo, Japan). The temperatures of the injection port and detector were both 250 °C. The column temperature was controlled as following: initial temperature was set at 50 °C for 1min, then, heated to 190 °C with the rate of 8 °C/min and held for 2 min, finally increased to 220 °C with the rate of 3 °C/min. The fatty acid species was identified using the retention time of the FAME standard solution and quantified based on relative peak area. The triplicate measurements for each sample were performed. 

### 3.3. Thermal Behavior and Crystallization at Different Cooling Rates by DSC

The thermal properties of the samples were measured using a DSC (Texas Instruments, Diamond-1, PerkinElmer, Dallas, TA, USA) with a Refrigerated Cooling System (Texas Instruments, Diamond-1, PerkinElmer, Dallas, TA, USA). Nitrogen was used to purge the thermal analysis system at a flow rate of 1 mL/min. The melted sample (4–5 mg) was weighed into an aluminum pan and sealed. An empty hermetically sealed aluminium sample pan was used as the reference. 

In recording the cooling and melting curves, the samples were first rapidly heated to 80 °C with the rate of 30 °C/min from room temperature and held for 10 min to ensure that the fats were totally melted and all the nuclei were destroyed. Then, the samples were cooled to −20 °C at the cooling rate of 10 °C /min and held for 20 min to make them fully crystallized, then heated to 80 °C at the heating rate of 5 °C /min.

Prior to investigate the effect of the cooling rate on crystallization, samples were rapidly heated to 80 °C with the rate of 30 °C/min from room temperature to erase the crystallization memory, then the samples were cooled to −20 °C at a cooling rate of 1 °C/min, 5 °C/min, 10 °C/min, 20 °C/min, respectively. The cooling thermogram was recorded. 

For investigating melting profile of PS and PO under isothermal crystallization, the samples were heated at 80 °C for 10 min and then cooled (100 °C/min) to a preset temperature (−10 °C, 0 °C, 10 °C, 20 °C), and hold at that temperature for 50 min for crystallization, then heated to 80 °C at the heating rate of 5 °C/min. The melting thermogram was recorded.

### 3.4. Isothermal Crystallization and Crystallization Kinetics by pNMR

For further study the crystallization kinetics of PS and PO, isothermal crystallization of palm oil fractions were performed in a Bruker PC20 series pulsed nuclear magnetic resonance (pNMR) analyzer (Bruker, Mississauga, ON, Canada). A rapid cooling and an accurate temperature control were realized by a water bath (DC-3006, Xin Zhi Instrument Company, Ningbo, China) equipped with pNMR. The samples in the NMR tube were initially heated to 70 °C and held for 20 min to eliminate any thermal history before measurement, then crystallized respectively at −10 °C, 0 °C, 10 °C and 20 °C in the pre-equilibrated thermostatted water bath. The values of % solid fat content (SFC) were measured by NMR every minute. Each analysis was executed in duplicate.

The isothermal crystallization kinectics was qualified by Avrami equation which has been reported to successfully describe crystallization kinectics of various polymers and additives in the polymer industry [27]. The Avrami equation can give an indication of the nature of the crystallization growth process [30]. Isothermal Avrami kinetics is concerned with the overall crystallization, including nucleation and growth [31]. Its primary form is:

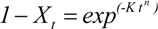
(1)
where *X_t_* is the relative crystallization at time *t*, *n* is the Avrami exponent, *K* is the Avrami rate constant. Equation 1 can be linearized by logarithmic transformation of [−ln(1-*X*)] and *t*, *K* and *n* were calculated from intercept and slope, respectively. In Equation 1, *n* expresses the power dependence on time of crystallization process, and can be assumed to be integer as well as non-integral value ranging from 0.5 to 4 [32]. The value of this exponent is directly related to the process governing the nucleation and growth mechanism [33]. Also, the *n* is a combination function of a time-dependent nucleation and number of potential growth dimensions [34]. The value of *X_t_* was calculated by integration of the isothermal crystallization as described by Henderson [35]:

*X*(*t*) = *SFC*(*t*)/*SFC*(∞) (2)

where *SFC(t)* describes the SFC as a function of time *t*, *SFC(∞)* is the limiting SFC as time approaches infinity. 

With better understanding the Avrami equation, modifications on the equation have been proposed. Considering that *K* was influenced by the magnitude of the nucleation process (*n*) [36], one modification is presented as:

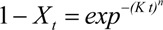
(3)


Half time of crystallization *(t_1/2_*) which expresses the magnitude of the Avrami constant [24] was calculated by Equation (4). It can be seen from Equation (4), the numerical value of *K* is directly related to the half time of crystallization:

*t*_1/2_ = (0.693/*K*)*^1/n^* (4)

### 3.5. Microscopy Analysis

Crystal morphology of the PS and PO were obtained under the same isothermal conditions utilized in the pNMR studies using a polarized microscope with Polarizing Microscophy (PLM) (DMRX, Leica, Solms, Germany) with a Canon A640 digital camera attached (Canon, Tokyo, Japan). The samples were melted at 80 °C and a drop was transferred onto a glass slide with the aid of a capillary tube. A cover slip was then placed parallel to the plane of the carrier glass and centered on the drop of sample to ensure uniformity and desirability of sample thickness. After this period, the slides were transferred to a place at −10 °C, 0 °C, 10 °C and 20 °C. Images were taken from three different visual fields at a magnification of 500. A single image was selected for the analysis [37].

## 4. Conclusions

PS and PO were separated from palm oil. The crystallization of PS and PO were closely associated with their chemical characteristics. PS and PO have the same kinds of fatty acids, but differ in content. Both of them showed quite similar exotherms. Compared to PO, exotherms of PS are sharper. The position and magnitude of the exotherms were dependent on the cooling rate. In respect to isothermal crystallization, when the crystallization temperature rose, the *n* value in Avrami equation increased, but *n* values were almost the same at the same temperature. The mechanism of nucleation changed from the high super-cooling instantaneous nucleation to low super-cooling sporadic nucleation. PS showed different crystal morphology compared to PO at the same temperature, also PS and PO formed different shapes of crystals at different temperatures. By controlling the crystallization process of PS and PO, its application in specific processes could become possible.
